# Seasonal Variations of *Spodoptera frugiperda* Host Plant Diversity and Parasitoid Complex in Southern and Central Benin

**DOI:** 10.3390/insects13060491

**Published:** 2022-05-24

**Authors:** Jeannette K. Winsou, Ghislain T. Tepa-Yotto, Karl H. Thunes, Richard Meadow, Manuele Tamò, May-Guri Sæthre

**Affiliations:** 1 Faculty of Biosciences (BIOVIT), Norwegian University of Life Sciences (NMBU), NO-1432 Ås, Norway; richard.meadow@nmbu.no; 2 Department for Invertebrate Pests and Weeds in Forestry, Horticulture and Agriculture, Norwegian Institute of Bioeconomy Research (NIBIO), NO-1431 Ås, Norway; karl.thunes@nibio.no; 3 Biorisk Management Facility (BIMAF), International Institute of Tropical Agriculture (IITA-Benin), Cotonou 08-01000, Benin; g.tepa-yotto@cgiar.org (G.T.T.-Y.); m.tamo@cgiar.org (M.T.); 4 Ecole de Gestion et de Production Végétale et Semencière (EGPVS), Université Nationale d’Agriculture (UNA), Kétou 43, Benin; 5 Plant Health Theme, International Centre of Insect Physiology and Ecology (*icipe*), Nairobi 30772-00100, Kenya; 6 Department for Climate, Energy and Environment, Section for Environment and Food Security, Norwegian Agency for Development and Cooperation (NORAD), NO-0257 Oslo, Norway; may-guri.saethre@norad.no

**Keywords:** fall armyworm, cultivated host plants, wild host plants, adapting parasitoids

## Abstract

**Simple Summary:**

The detection of fall armyworm (FAW) *Spodoptera frugiperda* (J.E. Smith, 1797) (Lepidoptera: Noctuidae) in 2016 attacking maize fields in central and west Africa indicated the need to increase the knowledge on the pest host plants and natural enemies adapting to it. A field survey was conducted for two years (from June 2018 to January 2020) to determine host plant and parasitoid records variations across seasons (maize growing and off-seasons) in selected sites in southern and central Benin. A total of eleven new host plant species were reported for the first time, including grasses. The survey revealed seven parasitoid species belonging to four families, namely Platygastridae, Braconidae, Ichneumonidae and Tachinidae, associated with FAW on maize and alternative host plants. The most abundant parasitoid species found was the egg parasitoid *Telenomus remus* (Nixon) (Hymenoptera: Platygastridae). The study provides crucial information for understanding the bioecology of the pest and for its long-term management using natural enemies.

**Abstract:**

Fall armyworm (FAW) *Spodoptera frugiperda* (J.E. Smith, 1797) (Lepidoptera: Noctuidae) was recorded for the first time in 2016 attacking maize fields in central and west Africa. Soon after, several other regions and countries have reported the pest in almost the entire sub-Saharan Africa. In the present study, we assumed that (i) a variety of alternative plant species host FAW, especially during maize off-season, (ii) a wide range of local parasitoids have adapted to FAW and (iii) parasitoid species composition and abundance vary across seasons. During a two-year survey (from June 2018 to January 2020), parasitoids and alternative host plants were identified from maize and vegetable production sites, along streams and lowlands, on garbage dumps and old maize fields in southern and partly in the central part of Benin during both maize growing- and off-season. A total of eleven new host plant species were reported for the first time, including *Cymbopogon citratus* (de Candolle) Stapf (cultivated lemon grass), *Bulbostylis coleotricha* (A. Richard) Clarke and *Pennisetum macrourum* von Trinius (wild). The survey revealed seven parasitoid species belonging to four families, namely Platygastridae, Braconidae, Ichneumonidae, and Tachinidae associated with FAW on maize and alternative host plants. The most abundant parasitoid species across seasons was the egg parasitoid *Telenomus remus* (Nixon) (Hymenoptera: Platygastridae). These findings demonstrate FAW capability to be active during the maize off-season in the selected agro-ecologies and provide baseline information for classical and augmentative biocontrol efforts.

## 1. Introduction

The Fall armyworm (FAW) *Spodoptera frugiperda* (J.E. Smith 1797) (Lepidoptera: Noctuidae) is one of the most economically important noctuid moth pests native to the Americas [1,2,3]. In Africa, the first outbreak of FAW was recorded in 2016 from central and west Africa in maize [4]. Soon after, several other regions and countries have reported the pest in almost the entire continent [5,6,7,8]. The estimated maize yield loss induced by FAW in Benin is 797.2 kg/ha, representing 49% of the commonly obtained average maize yield [9].

*S. frugiperda* is well known for its voracity on a range of crops and its periodical outbreaks in the Americas [1]. The larvae can feed on several cultivated or wild host plant species [1,10,11,12] but it is well known that maize is the prime host plant of FAW in its native range [1,6]. Despite most detections of FAW in Africa and elsewhere being chiefly on maize, it is anticipated that a range of plant species might host the pest during the maize off-season. Several of the plant families and species documented as host plants in the native range [1,12] are also common in the new invasion areas and may sustain the FAW populations in the absence of maize crops.

Sustainable FAW management strategies cannot be designed in areas of recent invasion without a prior in-depth assessment of the indigenous parasitoid biodiversity and related biological control potential. In extensive inventories in the Americas and the Caribbean Basin, more than 150 parasitoid species were found to be associated with FAW [13]. Among these, *Telenomus remus* (Nixon, 1937) (Hymenoptera: Platygastridae) was recognized as the most relevant naturally occurring egg parasitoid species. Initial inventory of the local fauna in Africa led to the discovery of *T. remus* [14] and other parasitoid species adapting to FAW, including *Chelonus bifoveolatus* Szépligeti, *Charops* sp., *Cotesia icipe* Fernandez-Triana and Fiaboe and *Coccygidium luteum* Brullé [15,16], but little is known about their seasonal variations. Therefore, the present study aimed at investigating in detail the seasonal variations of the host range and parasitoid diversity of FAW in Benin based on three assumptions (i) a variety of alternative plant species host FAW especially during maize off-season, (ii) a range of local egg and larval parasitoids have adapted to FAW and (iii) parasitoid species composition and abundance vary across seasons.

## 2. Materials and Methods

### 2.1. Study Sites

**Figure 1:**
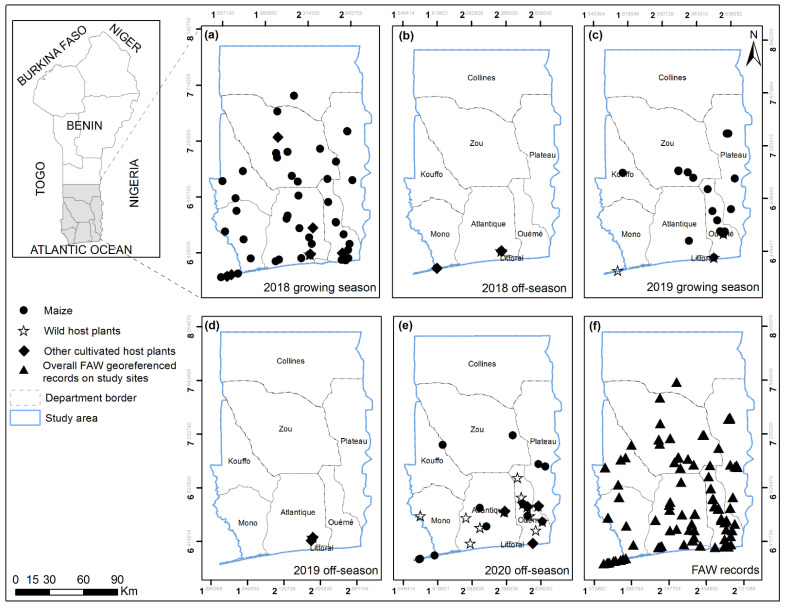
FAW occurrences on maize and alternative host plants during (**a**,**c**) growing seasons and (**b**,**d**,**e**) off-seasons for the years 2018, 2019 and 2020, and (**f**) overall FAW georeferenced records on study sites in southern and central Benin.

The study area is southern Benin and a few locations in the central parts of the country (Figure 1). The climate of southern Benin is characterized by two dry seasons (from December to February and August) and two rainy seasons (from March to July and September to November), respectively. In the central part of the country, there is one main rainy season from June to September and one dry season from October to May. The rainy season and the dry season match with maize’s growing and off-seasons, respectively. The survey sites were in all southern Benin departments, namely Atlantique, Kouffo, Mono, Littoral, Oueme, Zou and Plateau, but restricted to Collines department in central Benin. In total, 40 localities (year 2018), 23 localities (year 2019) and 25 localities (year 2020) were surveyed for both FAW host plant range and parasitoid complex records (Figure 1).

### 2.2. Field Surveys

Maize production sites and wet agroecosystems such as vegetable production sites, plants along streams and lowlands, plants on garbage dumps and old maize fields were selected randomly for sampling in southern and partly in central Benin during maize growing and off-season. Any infested plant (cultivated or not) with symptoms of FAW-like damage (window panes, frass) and all suspected host plants having FAW larvae or egg-masses were collected and brought to the laboratory for further identification. The sample size target was set to 100 plants to be sampled per hectare. However, because most fields visited belonged to smallholder farmers, it was uncommon to find big farms. In addition, few wild host plants were encountered harboring fall armyworm. Therefore, the sample size was based on host plant presence and ranged from 10 to 100 plants and occasionally more on maize fields. The eggs were kept in Petri dishes and followed until they hatched. Larvae were kept in plastic boxes (4.2 cm diameter; 5.3 cm height) covered with muslin and perforated cover, and fed with sprouting maize until pupation. The pupae were kept in the same type of plastic boxes until emergence of the adult moths. Upon confirmation of the identity of *S. frugiperda* by morphological examination of the larvae or moths, the host plant samples were sent to taxonomists at the National Botanical Reference Center at the University of Abomey-Calavi (UAC-Benin) for identification. All FAW host plants’ georeferenced points are provided in File S1.

To detect the presence of parasitoids, FAW larvae and egg-masses were collected from any of the infested plants as described above and monitored in the lab. Egg-masses were put in Petri dishes and checked at two-day intervals in order to record any larval emergence. After four days of incubation, all unhatched eggs or egg-masses were kept aside and monitored for FAW egg parasitoid emergence. Larvae were fed with sprouting maize as described above, and checked regularly for any signs of parasitism. Parasitoid pupae were transferred to small cages until emergence. Specimens of the parasitoids collected on FAW eggs and larvae were shipped to the Natural History Museum in London for species-level identification. All FAW parasitoids’ presence records are available in Files S2–S8.

### 2.3. Data Analysis

The correlation between the occurrence of parasitoid species and seasonal variations was tested by performing the chi-squared test for association (Ho < 0.05; H1 > 0.05) using R version 1.3.1093.

## 3. Results

### 3.1. FAW Host Plants Range

The survey in southern and central Benin revealed 29 alternative host plant species of FAW, belonging to 10 families. We are excited to report 11 new host plant species compared to the latest records of Montezano et al. [12] (Table 1). Regardless of season, the most abundant host plant families included Poaceae, Cyperaceae and Amaranthaceae with 13, 5 and 3 species, respectively. Ten and nineteen of the records comprised other cultivated and wild host species, respectively (Table 1). Most of the alternative cultivated host plants were recorded in Sèmè and Abomey-Calavi (Table 1), which harbor major vegetable production sites. The highest records of wild host plants were in Abomey-Calavi, but the reason for this is unclear (Table 1).

**Figure 2:**
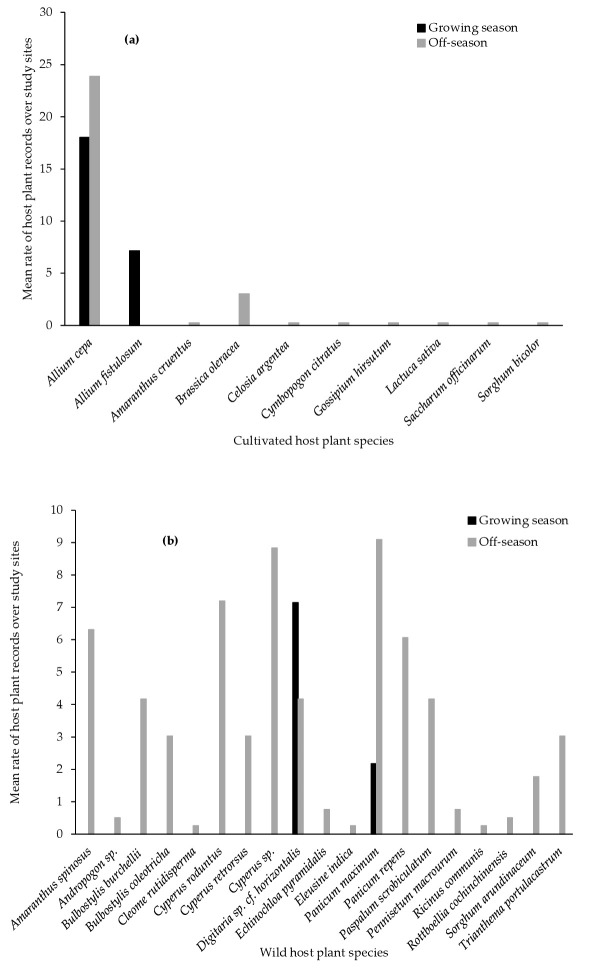
FAW host plant (**a**) cultivated crop and (**b**) wild species records over all study sites. The rate of host plant records for each study site is obtained by dividing the number of plants infested with FAW by the number of sites surveyed.

Not all plant species were found associated with FAW eggs or larvae across seasons (Figure 1 and Figure 2). Our findings revealed that the numbers of recorded alternative FAW host plants whether cultivated or wild increased during the observation period (Figure 1).

Onion (*Allium cepa* L.), Welsh onion (*Allium fistulosum* L.) and cabbage (*Brassica oleracea* L.) were the cultivated plant species most frequently found associated with FAW (Figure 2a). Onion was recorded with FAW during both maize growing and off-season, while *A. fistulosum* and *B. oleracea* were only found with FAW during off-season. FAW association with wild host plants was also season-dependent. Most of the wild host plants were recorded during off-season (Figure 2b). The five most important wild host plant species were: *Amaranthus spinosus* L., *Cyperus roduntus* L., *Cyperus* sp., *Digitaria* cf. *horizontalis* Willdenow and *Panicum maximum* Jacquin. Only *D*. cf. *horizontalis* and *P. maximum* hosted FAW during both maize growing and off-season. Overall FAW host plant records were higher in 2020 off-season compared to 2018 and 2019 off-seasons (Figure 1). FAW was found associated with maize during 2018 and 2020 off-seasons. Conversely, wild host plants were recorded in both 2018 and 2019 growing seasons.

### 3.2. FAW Parasitoid Complex

**Figure 3:**
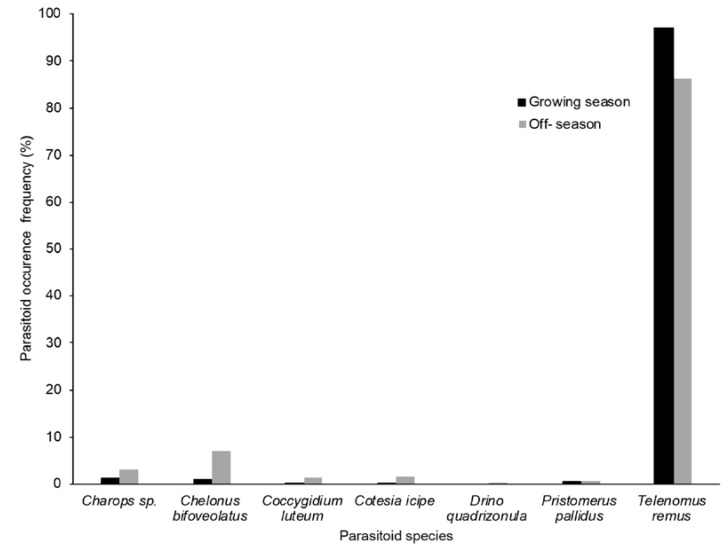
Occurrence of parasitoid species recorded in maize growing and off-seasons in southern and central Benin. The percentage of parasitoid frequency was calculated by dividing the number of the parasitoid species records by the total records of all parasitoid species.

Seven parasitoid species belonging to four families were collected: *Telenomus remus* Nixon (Hymenoptera; Platygastridae), *Chelonus bifoveolatus* Szépligeti, *Coccygidium luteum* (Brullé), *Cotesia icipe* Fernandez-Triana and Fiaboe (Hymenoptera; Braconidae), *Pristomerus pallidus* (Kriechbaumer), *Charops* sp. (Hymenoptera; Ichneumonidae) and *Drino quadrizonula* (Thomson) (Diptera; Tachinidae) (Table 2). The collections encompassed one egg parasitoid (*T. remus*), one egg-larval parasitoid (*Ch. bifoveolatus*) and the remaining five larval parasitoids representing 95; 2 and 3% of the entire parasitoid material collected, respectively (Figure 3).

**Figure 4:**
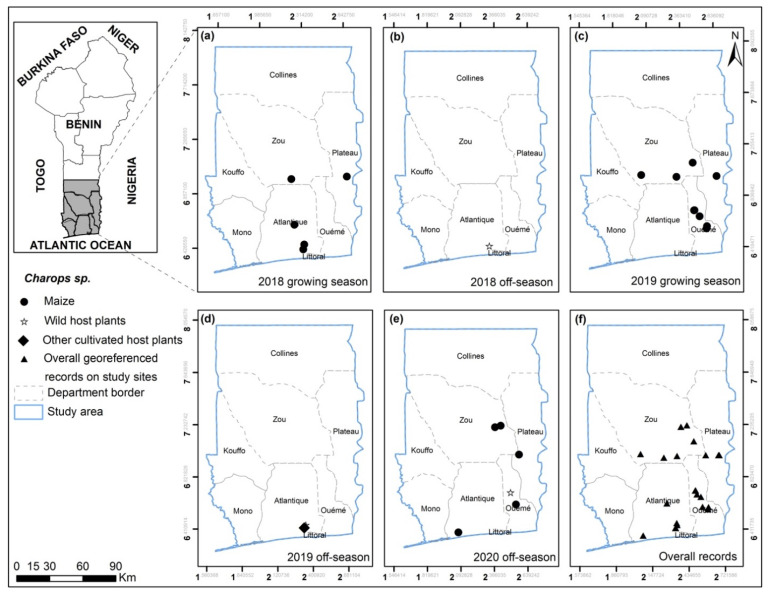
*Charops* sp. georeferenced records on maize, wild and other cultivated host plants for the years 2018, 2019 and 2020 on the study sites for growing (**a**,**c**) and off-seasons (**b**,**d**,**e**) in southern and central Benin (**f**).

**Figure 5:**
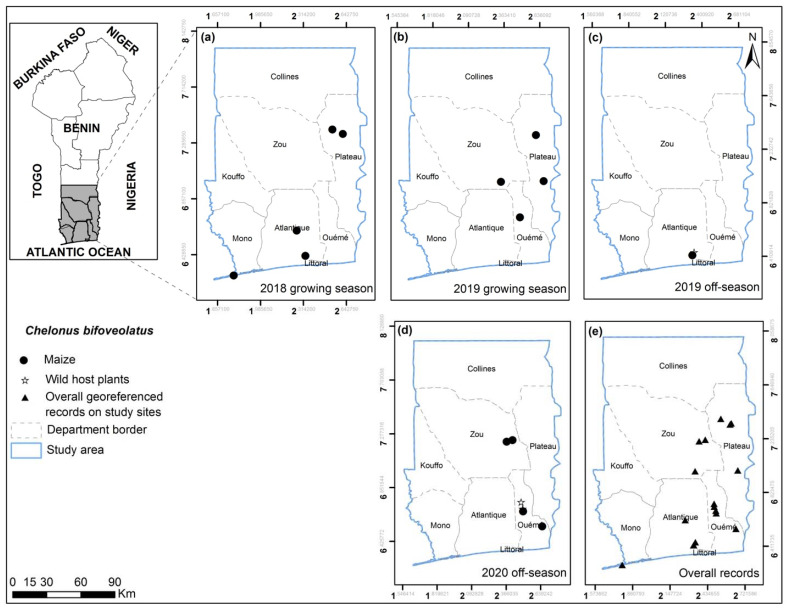
*Chelonus bifoveolatus* georeferenced records on maize, wild and other cultivated host plants for the years 2018, 2019 and 2020 on the study sites for growing (**a**,**b**) and off-seasons (**c**,**d**) in southern and central Benin (**e**). No *C. bifoveolatus* were found in the 2018 off-season.

**Figure 6:**
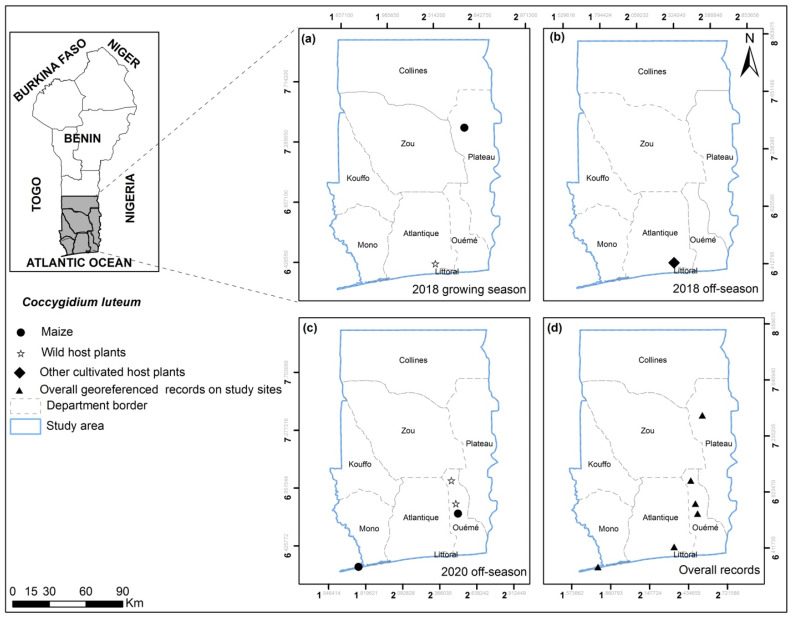
*Coccygidium luteum* georeferenced records on maize, wild and other cultivated host plants for the years 2018 and 2020 on the study sites for growing (**a**) and off-seasons (**b**,**c**) in southern and central Benin (**d**). No *C. luteum* were found in 2019.

**Figure 7:**
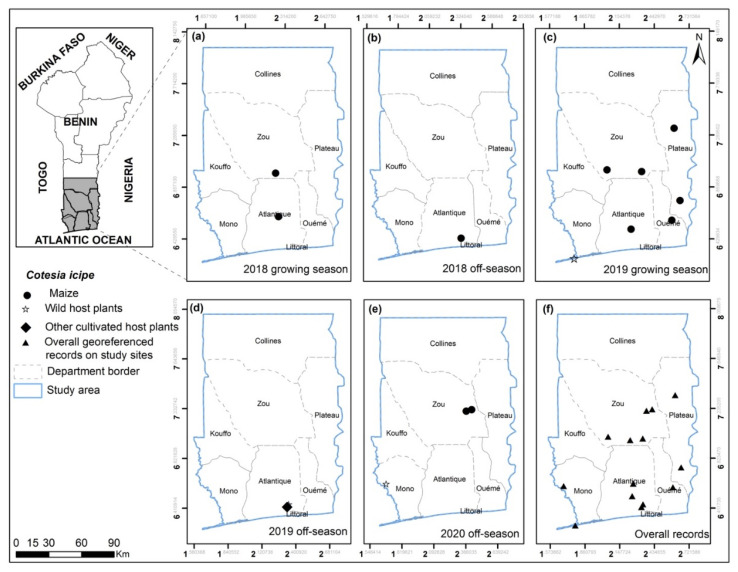
*Cotesia icipe* georeferenced records on maize, wild and other cultivated host plants for the years 2018, 2019 and 2020 on the study sites for growing (**a**,**c**) and off-seasons (**b**,**d**,**e**) in southern and central Benin (**f**).

**Figure 8:**
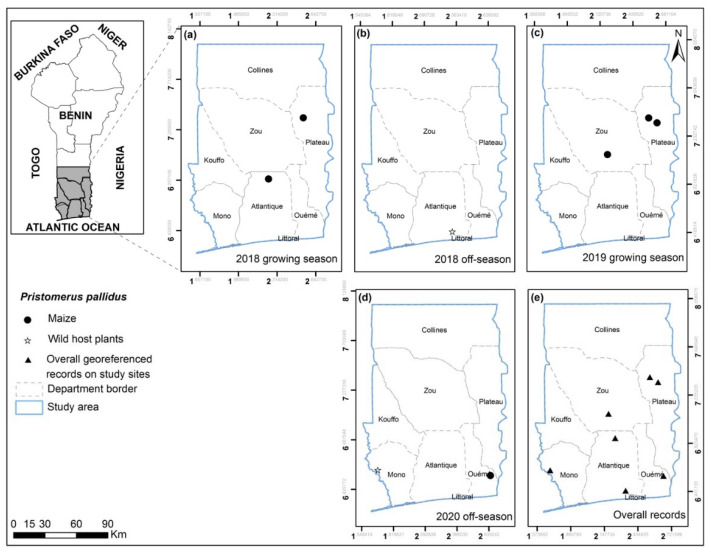
*Pristomerus pallidus* georeferenced records on maize, wild and other cultivated host plants for the years 2018, 2019 and 2020 on the study sites for growing (**a**,**c**) and off-seasons (**b**,**d**) in southern and central Benin (**e**). No *P. pallidus* were found in the 2019 off-season.

**Figure 9:**
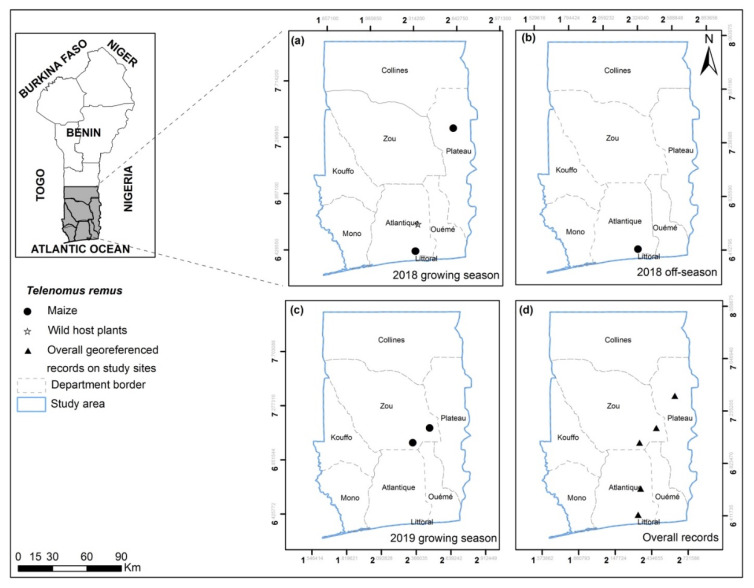
*Telenomus remus* georeferenced records on maize, wild and other cultivated host plants for the years 2018 and 2019 on the study sites for growing (**a**,**c**) and off-seasons (**b**) in southern and central Benin (**d**). No *T. remus* were found in the 2019 or 2020 off-season.

*Charops* sp (Figure 4) was recorded in Atlantique, Oueme, Plateau and Zou. *Chelonus bifoveolatus* (Figure 5) was also found in the same areas and in one more department (Mono). As for *Co. luteum* (Figure 6), *Co. icipe* (Figure 7) and *P. pallidus* (Figure 8), they were all discovered in Atlantique, Mono, Oueme and Plateau. In addition, *Co. icipe* and *P. pallidus* were found in Zou. The egg parasitoid *T. remus* (Figure 9) was recorded in Atlantique, Plateau and Zou while the larval parasitoid *D. quadrizonula* was identified in Atlantique only. Overall, FAW parasitoids were found in all study sites except in Kouffo, Collines and Littoral departments. Pearson’s chi-squared test showed a positive correlation between the maize season and the occurrence of parasitoid species (chi-square = 188.81; df = 6; *p* = 2.2 × 10^−16^).

All collected parasitoid species were associated with FAW larvae or egg-masses collected on both maize crops and wild host plant species (Table 3) except *D. quadrizonula* which was found in 2020 on maize only (Table 2) in one location of southern Benin. No *D. quadrizonula* parasitoids were found in 2018 and 2019. The egg parasitoid *T. remus* was recorded on *Panicum. maximum*, while the egg-larval parasitoid *Ch. bifoveolatus* was recorded on *Bulbostylis coleotrica*, *P. maximum* and *Andropogon* sp. The larval parasitoids *Charops* sp., *Co. luteum* and *Co. icipe* were also collected on onion and wild host plant species. These larval parasitoids were collected on *D*. cf. *horizontalis*, *B. coleotrica* and *Andropogon* sp. (*Charops* sp.); *D*. cf. *horizontalis*, *Sorghum arundinaceum, Cyperus* sp. and *P. maximum* (*Co. luteum*); *A. spinosus*, *B coleotrica* and *S. arundinaceum* (*Co. icipe*). *Pristomerus pallidus* was recorded on the wild plant species *C. roduntus* and *S. arundinaceum*.

## 4. Discussion

### 4.1. FAW Host Plant Range

The record of 29 alternative host plant species of FAW belonging to 10 families in southern and central Benin is the first intensive study of the FAW host plant range in Benin. The current report of 29 host plants is far fewer than the list of 180 and 353 species recorded by Casmuz et al. [1] and Montezano et al. [12] in the Americas, respectively. We are nevertheless excited to report 11 new host plant species compared to the latest records of Montezano et al. [12] (Table 1). However, our survey was limited to a two-year period, just a few years after first detection of the pest, and to selected localities in southern and central Benin. Therefore, we cannot exclude the possibility that FAW could become adapted to more host plants in the near future, and that some plant species may have been overlooked. The higher records of FAW host plants in 2020 compared to the 2018–2019 off-seasons can be a function of the natural spread of the pest. This could also be explained by the pest ability to adapt to more host plants with time in order to expand the resource food web with increasing pest populations post detection. However, the numbers of sites visited were not the same across seasons and years, there were higher in 2020 than in previous years. The records of FAW on maize during off-seasons (2018 and 2020) occurred in irrigated vegetable production areas where maize is often planted for dual purpose, i.e., diversification of food crops and fence plant. Most of the wild host plants found with FAW during off-seasons also had the pest in growing seasons (2018 and 2019).

Our study demonstrates that a variety of alternative plant species host FAW during maize off-season and explains why important pest infestation levels are commonly observed on maize crops after long off-season periods. The results are in agreement with earlier reports that FAW has a flexible host plant range which plays an important role in the long-term evolutionary survival of the pest [17]. The present corroborates previous observations that FAW, without a diapause mechanism, has developed survival strategy by feeding and maintaining its populations on alternative host plants [1]. FAW sustains its offspring on cultivated and wild alternative host plants (a high number of grasses) until the next maize growing season which clearly has implications for pest management.

The potential of grasses as oviposition sites for FAW supports the theory that *S. frugiperda* prefers C4 plants including maize as opposed to C3 plants [18,19]. This might be explained by the nutritional quality of C4 plants, which best fits the needs of the pest, compared to hosts from other botanical families [17,20]. Nevertheless, the question remains whether FAW is able to complete its whole life cycle on plants such as grasses that are weeds or that grow randomly. It is well known that FAW is highly voracious with the potential to attack different plant organs and then become either defoliator, cutter, granivore or borer [21]. FAW has the ability to move to nearby plants by crawling or by ballooning through secreted silks [1]. Therefore, the pest could complete its life cycle by moving to grasses.

### 4.2. FAW Parasitoid Complex

Seven parasitoid species belonging to four families were collected in this study. This report partly corroborates earlier findings in Benin by Agboyi et al. [15] who recorded an additional egg parasitoid *Trichogramma* sp. (Hymenoptera: Trichogrammatidae) on FAW. Data analysis revealed that there is a correlation between the season (maize growing and off-season) and the occurrence of parasitoid species recorded (Table 3). This may be a partial explanation of variations exhibited across different sampling efforts, while also considering that the geographic scope of those surveys are sometimes different. Moreover, the parasitoids recorded during the maize growing season were far more abundant than in the absence of maize. This could be due to the fact that the host is more abundant on maize crops than during off-seasons. Similar variations have been observed on other group of parasitoids [22].

All FAW parasitoids associated with FAW eggs and larvae collected on maize were also recorded on some of the alternative plant species surveyed except *D. quadrizonula* found on maize only.

Our results support model predictions on the capability of FAW and parasitoids to survive on most of the habitats in the area already heavily invaded by FAW and those potentially at risk [23]. The findings suggest some similarities in the plant volatiles induced by FAW damage on the prime host plant and those induced on alternative host plants. The plant volatiles emitted by FAW alternative host plants may play an important role for the parasitoids, possibly as attractant cues for the parasitism of FAW [24].

This work and earlier investigations in the Americas and especially in the Caribbean basin [13,25] confirm that in the absence of maize several host plants, either cultivated or wild, can constitute a reservoir of a range of FAW parasitoids. Nevertheless, the proportion of collected parasitoids is by far higher on maize (97% in this study) than all other alternative hosts combined, similar to earlier observations [13]. A possible explanation for this occurrence is that maize, being the preferred plant species supports higher populations of the host insect (FAW), also attracts more FAW parasitoids. Large numbers of *T. remus* were collected throughout the survey which concurs to the great potential of the species for biological control programs against FAW [14,15,23].

It is noteworthy to mention that our results are in agreement with recent records of parasitoid species in the local fauna adapting to FAW [15]. However, because of the limited geographical scope of our study, we cannot exclude the possibility that other species might have been overlooked. Nonetheless, the present report shows that further biocontrol efforts in Africa should carefully consider the potential of locally available parasitoid species along with the introduction of exotic species from the area of origin of FAW in the Americas.

## 5. Conclusions

Our initial assumptions that a variety of alternative plant species host FAW during maize off-season and that a wide range of local parasitoids have adapted to FAW were valid. In addition, this work illustrates that parasitoid species composition and abundance vary across seasons. We report eleven new host plant species of FAW compared to the latest records. The eleven new host plant species are: *A. cruentus, C. argentea, C. rutidosperma, B. burchellii, B. coleotricha, P. repens, C. citratus, E. pyramidalis, P. maximum, P. scrobiculatum* and *P. macrourum.* Seven parasitoid species belonging to four families were also collected during the study. *T. remus* was the most abundant and frequent parasitoid species found attacking FAW. Our results are in agreement with other records of parasitoid species in the local fauna adapting to FAW elsewhere in Africa, and highlight the potential of native species in the control of FAW.

## Figures and Tables

**Table 1:** FAW host plant species recorded in southern and central Benin. The eleven new host plant species records are in bold.

Host Plant Type	Family Name	Scientific Name	Sites of Collection
Cultivated	Amaranthaceae	***Amaranthus cruentus* L.**	Sèmè
		***Celosia argentea* L.**	Azowlissè
	Amaryllidaceae	*Allium cepa* L.	Abomey-Calavi, Agoué, Grand-Popo, Sèmè, Zinvié
		*Allium fistulosum* L.	Abomey-Calavi
	Asteraceae	*Lactuca sativa* L.	Sèmè
	Brassicaceae	*Brassica oleracea* L.	Abomey-Calavi
	Malvaceae	*Gossypium hirsutum* L.	Dan
	Poaceae	***Cymbopogon citratus* (de Candolle) Stapf**	Sèmè
		*Saccharum officinarum* L.	Zinvié
		*Sorghum bicolor* (L.) Mönch	Sakété
Wild	Aizoaceae	*Trianthema portulacastrum* L.	Sèmè
	Amaranthaceae	*Amaranthus spinosus* L.	Grand-Popo, Sèmè
	Cleomaceae	***Cleome rutidosperma de Candolle***	Azowlissè
	Cyperacea	***Bulbostylis burchellii* (Ficalho and Hiern) C.B. Clarke**	Abomey-Calavi
	Cyperacea	***Bulbostylis coleotricha* (A. Richard) Clarke**	Abomey-Calavi
		*Cyperus rotundus* L.	Abomey-Calavi
		*Cyperus retrorsus* Chapman	Abomey-Calavi
		*Cyperus* sp.	Adjohoun, Tori-Avamè
	Euphorbiaceae	*Ricinus communis* L.	Zinvié
	Poaceae	*Andropogon* sp.	Adjohoun, Athiémé
		*Digitaria sp.* cf. *horizontalis* Willdenow	Abomey-Calavi
		***Echinochloa pyramidalis*** **(Lamarck) Hitchcock and Chase**	Zinvié, Kpomassè
		*Eleusine indica* (L.) Grtner	Tori-Avamè
		***Panicum maximum* Jacquin**	Abomey-Calavi, Zinvié, Misserete, Bonou, Dangbo, Adjohoun
		***Panicum repens* L.**	Abomey-Calavi
		***Paspalum scrobiculatum* L.**	Abomey-Calavi
		***Pennisetum macrourum*** **von Trinius**	Adjohoun, Azowlissè
		*Sorghum arundinaceum* (Desvaux.) Stapf.	Aguégués, Athiémé, Avrankou, Tori-Avamè, Ouidah, Bonou
		*Rottboellia cochinchinensis* (Loureiro) W.D.Clayton	Sakété, Athiémé

**Table 2:** FAW parasitoids complex associated with alternative host plants and maize in southern and central Benin.

Order, Family and Species	Locality	Host Plant	FAW Stage Collected
Hymenoptera: Platygastridae			
*Telenomus remus* Nixon	Ab-Cal; Aid (Ket); Zinv; Ouin; Zog	Maize; *Panicum maximum*	Egg
Hymenoptera: Braconidae *Chelonus bifoveolatus* Szépligeti	GP; Ag; Ket; Aid (Ket); Glo; Ab-Cal; Adj; Kpa; Pob; Zog; Avr; Zag; Azo	Maize; *Bulbostylis coleotrica*; *Panicum maximum*; *Andropogon* sp.	Larva; Egg
*Coccygidium luteum* (Brullé)	Ket; Ab-Cal; Bon; Adj; Azo; GP	Maize; *Digitaria* cf. *horizontalis; Allium cepa; Sorghum arundinaceum; Cyperus* sp.; *Panicum maximum*	Larva
*Cotesia icipe* Fernandez-Triana and Fiaboe	Glo; Mas; GP; Dra; Kpa; Sak; Mis; Ab-Cal; Zog; Zag; Ath	Maize; *Amaranthus spinosus*; *Bulbostylis coleotrica*; *Allium cepa*; *Sorghum arundinaceum*	Larva
Hymenoptera: Ichneumonidae *Charops* sp.	Glod; Mas; Pob; Ab-Cal; Dan; Adj; Mis; Pob; Azo; Ak-Mis; Ouin; Zog; Ab-Cal; Azo; Zag; All; Dan; Adja; Adj	Maize; *Digitaria* sp.; *Allium cepa; Bulbostylis coleotrica; Andropogon* sp.	Larva
*Pristomerus pallidus* (Kriechbaumer)	Ket; Hou (Ag); Ab-Cal; Kpa; Zog; Ath; Avr	Maize; *Cyperus roduntus*; *Sorghum arundinaceum*	Larva
Diptera: Tachinidae *Drino quadrizonula* (Thomson)	Ab-Cal	Maize	Larva

Aidjedo (Ketou): Aid (Ket); Abomey-Calavi: Ab-Cal; Zinvié: Zinv; Ouinhi: Ouin; Zogbodomey: Zog; Glo: Glo; Adjohoun: Adj; Kpankoun: Kpa; Pobè: Pob; Avrankou: Avr; Zagnanado: Zag; Grand-Popo: GP; Agoué: Ag; Ketou: Ket; Azowlissè: Azo; Bonou: Bon; Sakete: Sak; Massi: Mas; Drabo: Dra; Missérété: Mis; Athiémé: Ath; Glodjigbé: Glod; Dangbo: Dan; Akpro-Missérété: Ak-Mis; Allada: All; Adjawèrè: Adja; Houègbo (Agon): Hou (Ag).

**Table 3:** FAW parasitoids recorded on the pest eggs and larvae collected on alternative host plants.

Order, Family and Species	Alternatives Host Plants	Number of Collected Specimens
Hymenoptera: Platygastridae *Telenomus remus*	*Panicum maximum*	100
Hymenoptera: Braconidae *Chelonus bifoveolatus*		
	*Bulbostylis coleotrica*	1
	*Panicum maximum*	2
	*Andropogon* sp.	1
*Coccygidium luteum*	*Digitaria* cf. *horizontalis*	1
	*Allium cepa*	1
	*Sorghum arundinaceum*	1
	*Cyperus* sp.	1
	*Panicum maximum*	2
*Cotesia icipe*	*Amaranthus spinosus*	1
	*Bulbostylis coleotrica*	4
	*Allium cepa*	1
	*Sorghum arundinaceum*	1
Hymenoptera: Ichneumonidae	*Digitaria* sp.	1
*Charops* sp.	*Allium cepa*	3
	*Bulbostylis coleotrica*	3
	*Andropogon* sp.	3
*Pristomerus pallidus*	*Cyperus roduntus*	1
	*Sorghum arundinaceum*	1

## Data Availability

The data presented in this study are available in article or Supplementary Materials.

## Supplementary Materials

The following supporting information can be downloaded at: https://www.mdpi.com/article/10.3390/insects13060491/s1, File S1: FAW host plant records; File S2: *Charops* sp. records; File S3: *Chelonus bifoveolatus* records; File S4: *Coccygidium luteum* records; File S5: *Cotesia icipe* records; File S6: *Drino quadrizonula* records; File S7: *Pristomerus pallidus* records; File S8: *Telenomus remus* records.
